# Supplementary material for: Chromothripsis during telomere crisis is independent of NHEJ, and consistent with a replicative origin
Source: Genome Res. 2019 May;29(5):737–49. doi: 10.1101/gr.240705.118 (PMC6499312; doi:10.1101/gr.240705.118)
Supplement: Supplemental Material [file supp_gr.240705.118_Supplemental_file_1.zip › contigs/annotated_contigs/DB45/contig.3.DB45_length_944_mean_cov_7.62711864407.docx]

**DB45_length_944_mean_cov_7.62711864407**

```
GATTGTTTTAAGGATTACATAAGGTATGCTATGCAGGTTAAGTATTTGTCTCACAGTAGTCTTGCATTATTATTGTAAAAGTTAGAAAA
 >chr4:44208857-44209133 + E=4e-154
TAAAAAAATAACCATTCAAAATGAACATTTACTATTTGTTGGGTCCTTTTAAGAGCTGACTTTACAAGTTGTGAGATAGTATATTTGTG

GCCAAGTATCAGATAAGCAACCTAATACTTGGAGATGTCAAACGTCTTTGTTCTAACACTTGTGCTGTGACTTTCCAGCTGTGTAACCT

TGAACAAGT|G|TTCGTTAAACACATTGCAATTACTGTATTACAACAATGGAAAAGAATAGTAGATAATTGCTGGGGAATTTGAGAAAA
           >chr4:44209898-44210253 - E=1e-201
TTTCTAAGAAAGTTAATTTGAACCCCTTCTTAAAGGCTGTGGTCACCTTTTGTACCCAAAGAGGAAAACTGTGGTGGACATAGGGTTTC

TTAGCCTATCACAATCTCCTGGAATTTCTTCAGCACTTCTCTCTTGAGCTCCAGAAGTTCTGATGTCACCAAATAGGTCAAGTGGGATC

ATTTCAGCTAGGTCTTTGCATCTTTGATTCTCATCCCTGCTTACTCTGCCTGTGTTTATATTGCTAGCAACTCAGCAGCAAGCCATTCC

CCATGCTT|GTA|ATTACCTGAGAGTACAGCCACAGGGATCTTCAGAGAATGGCAGCACCCTCAGACTGCATCCTAATGAGTAGCCTTT
         >chr4:44210369-44210684 + E=2e-177
TAATCTTCACCTCCATCTCTTATCACTACTTCCGTAACACGAAGCATTTCCTAATTAGGATCATCATATCAGGTGACAAGATAGAATAC

AAATTATATTGAAAATCACAAAATAATACCAAATAAGAAGTTTGGTATGCTCCAATAGTACAATGTATGCAAACTTTTCACCCTAAAAG

AAAATGATCTTCTTGTCCAGGAGTTGGAATAATTGGGCTACAGAGACTGAAGTCCTAT
```
